# Transcranial Magnetic Stimulation Meets Virtual Reality: The Potential of Integrating Brain Stimulation With a Simulative Technology for Food Addiction

**DOI:** 10.3389/fnins.2020.00720

**Published:** 2020-07-14

**Authors:** Chiara Stramba-Badiale, Valentina Mancuso, Silvia Cavedoni, Elisa Pedroli, Pietro Cipresso, Giuseppe Riva

**Affiliations:** ^1^Applied Technology for Neuro-Psychology Lab, Istituto Auxologico Italiano, Istituto di Ricovero e Cura a Carattere Scientifico, Milan, Italy; ^2^Department of Psychology, E-Campus University, Novedrate, Italy; ^3^Department of Psychology, Catholic University of the Sacred Heart, Milan, Italy

**Keywords:** food addiction, TMS, virtual reality, exposure therapy, craving

## Abstract

The aim of this perspective is to propose and discuss the integration of transcranial magnetic stimulation (TMS) over the dorsolateral prefrontal cortex with virtual reality (VR) food exposure for therapeutic interventions for food addiction. “Food addiction” is a dysfunctional eating pattern which is typically observed in eating disorders (ED) such as bulimia nervosa and binge eating disorder. Food addiction has been compared to substance use disorder due to the necessity of consuming a substance (food) and the presence of a dependence behavior. In recent years, VR has been applied in the treatment of ED because it triggers psychological and physiological responses through food exposure in place of real stimuli. Virtual reality-Cue exposure therapy has been proven as a valid technique for regulating anxiety and food craving in ED. More, TMS has been proven to modulate circuits and networks implicated in neuropsychiatric disorders and is effective in treating addiction such as nicotine craving and consumption and cocaine use disorder. The combination of a simulative technology and a neurostimulation would presumably provide better improvement compared to a single intervention because it implies the presence of both cognitive and neuropsychological techniques. The possible advantage of this approach will be discussed in the perspective.

## Introduction

“Food addiction” is a dysfunctional eating pattern typically observed in eating disorders (ED) such as bulimia nervosa (BN) and binge eating disorder (BED; Meule and Gearhardt, 2014). It has been compared to substance use disorder (SUD) due to the necessity of consuming a substance (food) and the presence of a dependence behavior (Meule and Gearhardt, 2014).

Transcranial magnetic stimulation (TMS) might be effective in modulating circuits and networks implicated in neuropsychiatric disorders and in treating addictions, such as nicotine craving/consumption and cocaine use disorder (Diana et al., 2017; Su et al., 2017; Ma et al., 2019; Steele et al., 2019).

In recent years, virtual reality (VR), a new technology capable of simulating real life scenarios (Riva et al., 2019b), has been applied in the treatment of ED because it can trigger psychological and physiological responses through virtual food exposure in place of real stimuli (Koskina et al., 2013; Pla-sanjuanelo et al., 2015). Virtual reality-based Cue exposure therapy (VR-CET) represents a valid technique for regulating anxiety and food craving in weight and EDs (Ferrer-garcia et al., 2017).

The combination of a simulative technology and a neurostimulation would presumably provide better improvement compared to a single intervention, because it copes with both cognitive-emotional and brain mechanism underpinning EDs. The possible advantages of this approach will be discussed in this perspective.

## Food Addiction

For decades, the idea that specific kind of foods may have an addiction potential and that overeating may be an addicted behavior has been discussed (Meule and Gearhardt, 2014).

Randolph (1956) first introduced the term “food addiction” referring to the reaction of overeating specific kind of foods followed by malaise, addiction, and long-term negative physical consequences as obesity (Randolph, 1956).

In 2009 The Yale Food Addiction Scale (YFAS; Gearhardt et al., 2009) was invented based on the Diagnostics and Statistical Manual of Mental Disorders fourth edition (DSM IV-TR) criteria for substance-related and addictive disorders (American Psychiatric Association, 2000). Food addiction was thus defined by the presence of three or more of the following symptoms: (1) a larger amount of food taken for a longer period than intended, (2) persistent desire or repeated unsuccessful attempts to reduce or stop intake of certain foods, (3) much time/activity to obtain, use, recover from eating certain foods, (4) important social, occupational, or recreational activities given up or reduced as a result of symptoms, (5) continued intake of the food(s) despite knowledge of adverse consequences, (6) tolerance, (7) withdrawal. In 2016, according to the DSM 5 (American Psychiatric Association, 2013) YFAS was changed into YFAS 2.0 (Gearhardt et al., 2016) including four additional symptoms: (8) continued use despite social or interpersonal problem, (9) failure to fulfill major role obligations, (10) intake of certain foods in physically hazardous situations, and (11) craving or strong desire/urge to eat certain foods.

Neurobiological studies revealed altered neural activity both in presence and absence of palatable food cues. For instance, when anticipating palatable foods, activity in left medial orbitofrontal cortex (OFC), left anterior cingulate cortex (ACC) and amygdala positively correlates with food addiction severity (Gearhardt et al., 2011). In addition, higher YFAS scores correlated with greater anticipatory activity of the left dorsolateral prefrontal cortex (DLPFC), which is involved in inhibition and reward control, due to its connections with ventral limbic circuitry (Wallis and Miller, 2003; Jauregui-Lobera and Martinez-Quinones, 2018). Instead, in absence of food exposure, YFAS scores correlate with ACC activity (Gearhardt et al., 2011). As in SUD, (Franken et al., 2004; Coullaut-Valera et al., 2014) food addiction is associated with abnormalities in the dopaminergic system (Davis et al., 2013, 2014) responsible of the reward system (Wang et al., 2011). Substance use disorder and food addiction share also some behavioral features including impulsivity, anxiety, depression symptoms, and attentional biases towards food-drug stimuli (Naish et al., 2018).

Food addiction prevalence appears increased in patients who suffer from BN and BED (Pursey et al., 2014) both characterized by compulsive episodes of disproportionate consumption of highly palatable foods together with a strong sense of loss of control. Three main elements define BED: preoccupation/anticipation, binge/intoxication, and withdrawal/negative effect (American Psychiatric Association, 2013). These three stages interact with each other, gradually becoming more intense and eventually leading to the pathological state known as addiction. Preoccupation and anticipation are known as craving, a physiological condition which, despite a state of satiety, determines food intake of desired food (Jauregui-Lobera and Martinez-Quinones, 2018).

Taken these results together, food addiction is reconcilable to a SUD due the joint dependence on a behavior (eating) and a substance (food) (Meule and Gearhardt, 2014).

## Transcranial Magnetic Stimulation

Transcranial magnetic stimulation is a non-invasive brain stimulation (NIBS) technique capable of modulating the neural activity of a targeted brain region. It relies on the induction of an electric field in the brain, delivered by a coil, which influences cortical excitability, by activating or deactivating neural networks (Rossi et al., 2009). Transcranial magnetic stimulation effects depend on frequency, intensity, number of pulses delivered, type of coil employed, and location of the stimulation (Diana et al., 2017). It can be distinguished between single-pulse TMS, in which one stimulus at a time is applied; paired-pulse TMS in which pairs of stimuli separated by an interval are applied and repetitive TMS (rTMS), in which pulses are delivered in trains (Pascual-Leone et al., 2000). Specifically, rTMS can be applied either as trains of high-frequency (more than 1 Hz), increasing cortical excitability, or trains of low-frequency (1 Hz or less), inhibiting the targeted region (Fitzgerald et al., 2006; Rossi et al., 2009). Usually, TMS treatments are compared to sham condition in which, by tilting the coil away from the scalp, the magnetic field does not reach cortical neurons, even though sound and scalp contact are similar to those experienced during active stimulation (Sandrini et al., 2011).

Over the past decades, NIBS is being increasingly employed in clinical practice (Brunoni et al., 2019). Both rTMS and transcranial direct stimulation (tDCS) have been primarily applied to patients suffering from depression reporting successful enhancement of mood (Wassermann and Zimmermann, 2012; Shin et al., 2015). Transcranial magnetic stimulation has also been employed as a promising alternative to pharmacological therapies, for several other psychiatric disorders which appear to be drug-resistant/non-responsive to medications including bipolar disorder (Xia et al., 2008), schizophrenia (Shi et al., 2014), obsessive-compulsive disorder (Berlim et al., 2013), anxiety disorder (Dilkov et al., 2017), panic disorder (Mantovani et al., 2013), post-traumatic stress disorder (Watts et al., 2012), and addiction disorder (Diana et al., 2017; Hauer et al., 2019; Steele et al., 2019).

According to the DSM-5, “addiction” is a chronic brain disorder characterized by the need of a substance or an engaging activity, determining a compulsive and uncontrollable behavior difficult to control, despite negative consequences (American Psychiatric Association, 2013). Neuroimaging studies proved that craving implies alterations in brain regions (Koob and Volkow, 2016; Diana et al., 2017) particularly in DLPFC and orbito-frontal cortices (Hayashi et al., 2013). Dorsolateral prefrontal cortex is involved in regulating craving effects, controlling response inhibition and self-regulatory (Brody et al., 2002; Mcbride et al., 2006). Indeed, high frequency rTMS delivered over this area reduced drugs, nicotine, or alcohol consumption (Diana et al., 2017; Su et al., 2017; Ma et al., 2019; Steele et al., 2019). Since neuromodulation have been found to manipulate DLPFC in favor of substance craving reductions (Brody et al., 2002; Mcbride et al., 2006; Liu et al., 2017; Su et al., 2017); some authors studied its efficacy also on food craving symptoms.

### TMS and ED

Recent studies have highlighted TMS effectiveness in treating eating and weight disorders including BN, obesity, and BED. For instance, Van Den Eynde et al. (2010) administered one rTMS or sham stimulation over the left DLPFC and reported a decrease in binge-eating episodes and in the urge to eat only in the active condition. More recently, Sutoh et al. (2016) assessed cerebral oxygenation, food craving and bulimic symptoms changes induced by a single rTMS session over the left DLPFC. A significant reduction in food craving was reported by subjective ratings and a significant decrease of cerebral oxygenation in the left DLPFC was observed following rTMS session. Kim et al. (2017) evaluated weight loss in obese patients following four rTMS sessions over the left DLPFC reporting significant weight loss, reduced body mass index and visceral adipose tissue. More recently, Kim et al. (2019) confirmed the same results also at 4-weeks follow-up proving long-term effects on eating consumptions. Rachid (2018) reviewed rTMS single and multiple sessions studies showing relative effectiveness in reducing both craving and ED symptoms. Promising results were reported also in a single case study of a woman with BED and major depression diagnosis (Baczynski et al., 2014): after 20 rTMS sessions over the left DLPFC the Binge Eating Scale (BES) and the Beck Depression Inventory (BDI) scores significantly decreased and the patient reported no binge eating episodes for that week.

These findings together emphasize the role of frontostriatal pathways and of the dopamine in ED, shedding light on the potential contribution of rTMS over DLPFC to correct these abnormalities.

## Virtual Reality

Virtual reality is a computer application by which humans interact with 3D computer-generated environments creating life-like contexts, involving various senses (Bohil et al., 2011). There are different degrees of immersion and interaction that modulate how the user experiments the feeling of being immersed in the VR (Slater, 2009; Cipresso et al., 2018); along with a sense of presence within the environments (Riva and Mantovani, 2014; Riva et al., 2018). Further, virtual stimuli can elicit reflexive responses similar to those produced by equivalent situations in real life (Meehan et al., 2002). These features contribute to the possibility to physically and emotionally interact within the environment: in particular Chirico et al. (2017) found that VR can effectively induce awe (e.g., the feeling of the view from the top of a mountain), usually difficult to reproduce in laboratory settings.

The combination of emotional, physical and mental interaction supports high motivation and engagement (Teo et al., 2016). More, another potential lies on the ability of VR to provide augmented feedback (e.g., auditory, visual, and kinesthetic), complimentary to the ones received through the sensory system. In this regard, VR therapy has been employed as a treatment for cognitive and motor dysfunction to improve neuroplasticity by engaging patients in multisensory training (Adamovich et al., 2009). In recent years, VR technologies have become widely used for the treatment of several disorders including post-traumatic stress disorder (Rizzo et al., 2011), pain management (Matamala-gomez et al., 2019), anxiety and depression (Mishkind et al., 2017; Zeng et al., 2018), neuropsychological deficits (Montana et al., 2019), and traumatic brain injury (Spreij et al., 2014).

### VR and ED

Virtual reality has also been employed in the field of EDs since 1990s (Ferrer-garcía and Gutiérrez-maldonado, 2012; Koskina et al., 2013; Ferrer-garcia et al., 2017).

In this regard, Riva et al. (2019a) argued that all EDs share a common “normative discontent” (Rodin and Larson, 1992) about the own body experience. Personal factors (e.g., body mass index, sex, age, and personality), interpersonal factors (e.g., parents and peers’ relations) and socio-cultural/economic environment (e.g., body model, ideal size, physical fitness, and athletic body) could determine a negative body representation.

Over the past thirty years, VR technologies have been used to explore the concept of body image (Riva et al., 1997; Perpiñá et al., 1999), its situation-dependent changes (Gutiérrez-Maldonado et al., 2010; Ferrer-garcía and Gutiérrez-maldonado, 2012), and to study emotional and behavioral responses to food cues exposition (Fett et al., 2009; Schienle et al., 2009). Riva et al. (2019a) identified three different randomized controlled trials that have shown, at one-year follow-up, that VR-CET had a higher efficacy than cognitive behavioral therapy (CBT) in preventing weight regain but not in better managing binge eating episodes in obese BED patients (Cesa et al., 2013; Marco et al., 2013; Manzoni et al., 2016). Moreover, a randomized study by Ferrer-garcia et al. (2017) showed a decrease in craving and anxiety symptoms after exposure to craved virtual food in virtual environments (i.e., kitchen, dining room, bedroom, and cafeteria) in BN and BED patients who were previously treated with classical CBT without significant outcomes. As assessed by self-report questionnaires, patients showed significant reductions in terms of binge and purge episodes, as well as the decrease of the tendency to engage in overeating episodes. In two additional studies, VR treatments reduced eating-related anxiety during and after exposure to virtual food, helping to disrupt the reconsolidation of adverse, food-related memories (Koskina et al., 2013; Pla-sanjuanelo et al., 2015).

Furthermore, based on the *Allocentric Lock Theory*^1^, VR plays a role on body image concept, helping to restore allocentric and egocentric representations (Riva and Mantovani, 2014). Finally, VR allows multisensory bodily illusions as well, such as the “Full Body Illusions” that offers illusory ownership over a virtual fake body able to temporarily correct the individual’s experience of distorted body shape and size (Keizer et al., 2016; Serino et al., 2016).

## The Clinical Application of NIBS and VR

The integration of NIBS and VR has been assessed in different clinical context. On one hand, NIBS is a promising treatment which targets neurophysiology, on the other hand, VR offers an ecological, controlled, and motivating environments tailoring different diseases and needs. Thus, different studies suggested that the combination of NIBS and VR could be more synergistic (Bagce et al., 2012; Kim et al., 2014) compared to stand-alone treatments for stroke rehabilitation. Massetti et al. (2017) reviewed their integration in both clinical (e.g., stroke survivors, children with cerebral palsy, and healthy population) showing positive results in terms of body sway, gait, recovery after stroke, pain management, vegetative reactions, improved learning, and performances with possible applications in neural rehabilitation. The authors supported neuromodulation potential in priming the brain prior to other therapies, enhancing clinical outcomes in neurological conditions even though information regarding its frequency and duration are needed. Subramanian and Prasanna (2018) conducted a meta-analysis on the suitability of NIBS-VR combination in post-stroke upper limb motor rehabilitation showing that effectiveness of NIBS varied depending on the stage of the stroke. Studies that employed in the sub-acute stage contralesional cathodal tDCS (Lee and Chun, 2014) and inhibited TMS (Zheng et al., 2015) showed greater improvements. Benefits from these studies might be related to a decrease of the transcallosal inhibition from contralesional to the ipsilesional hemisphere, which determined motor improvement (Bertolucci et al., 2018). Nevertheless, the generalizability of the findings is limited by the number and the heterogeneity of the studies included. Bassolino et al. (2018) induced embodiment for an artificial hand in a virtual environment through TMS over corticospinal tract, without tactile cues on the hand’s skin, typically used to ease rubber hand illusion. Authors argued that this illusory embodiment was determined by neuro-visual integration between TMS over primary motor cortex (and connections with sensory cortex) and hand twitches with visual VR feedback (Bassolino et al., 2018). This effect did not occur when sham TMS was delivered, suggesting that the temporal synchrony between active TMS and VR feedback determined the illusory embodiment.

Fewer studies investigated the combination of these technologies in mood and anxiety disorders. In 2015 Notzon et al. (2015) investigated the efficacy of intermittent theta burst stimulation (iTBS) on anxiety provoked by VR environment. On one hand, VR was effective in inducing anxiety arousal (provoked by virtual spiders) along with typical physiological activations [e.g., heart rate (HR), heart rate variability (HRV)]. On the other hand, iTBS did not provide significant results in subjective and psychophysiological reactions but could modulate HRV, in contrast to other studies that revealed rTMS anxiolytic potential (Lefaucheur et al., 2014; Zwanzger et al., 2009). Future studies should assess the effectiveness of repeated iTBS sessions for anxiety treatment.

Taken these results together, preliminary evidence suggest that employing multiple session of NIBS during the VR therapy might enhance the effects of VR or neuromodulation interventions alone (Teo et al., 2016). Nevertheless, this integrated approach has been employed only for specific disorders including anxiety, stroke and pain recovery and specific information in terms of the number of sessions, the intensity and the duration needed to obtain positive outcomes are lacking. For this purpose. we aim to address this issue by expanding the existing literature about NIBS and VR integration.

### A New Integrated Approach

This perspective draws on previous promising studies and aims to propose and discuss the integration of TMS over the DLPFC with VR-CET, for therapeutic interventions of food addiction. This integrated approach might allow the assessment and the monitoring of all the food craving variables (i.e., neuro-psycho-physiological, emotional, behavioral and cognitive) which are involved in food addiction (Ghi and Gutiérrez-maldonado, 2018).

#### Methods and Procedure

In this section we will propose a possible protocol aimed at assessing this new integrated approach.

The study will be performed in ED patients with at least 3 symptoms assessed by YFAS 2.0. Participants will be randomly assigned to the experimental condition which consists of eight sessions of active rTMS + VR-CET (*N* = 20) or to the control condition (*N* = 20) consisting in 8 sessions of sham rTMS + VR-CET. The sample size was estimated based on previous study (Kim et al., 2019) which gave 80% power to detect a significant *p* < 0.05 difference between groups.

Firstly, patients will undertake high-frequency rTMS or TMS sham sessions. TMS frequency will be determined by preliminary investigations. Active rTMS will be delivered over the left DLPCF because neuromodulation over this region has been associated with decreased food craving and ED symptoms and with increased dopaminergic activity.

**FIGURE 1:**
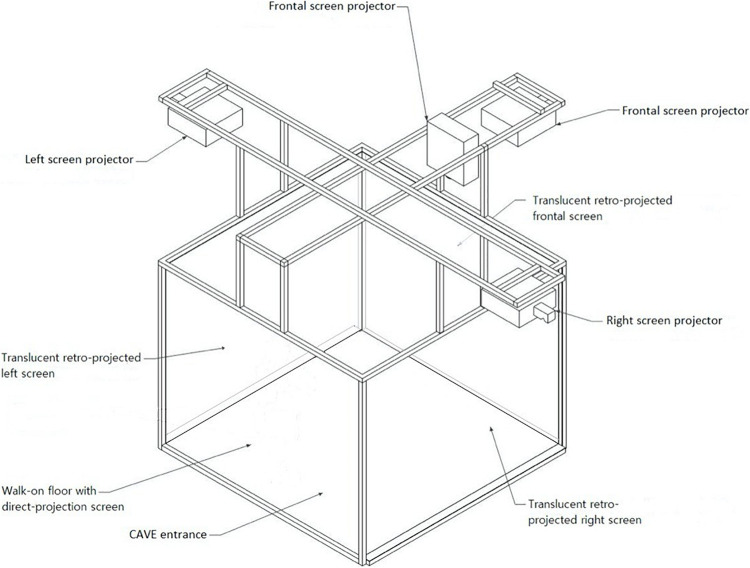
The CAVE is a 3-D immersive four-walled room in which the 3D visualization of the virtual scenario occurs through four stereoscopic projectors. There are three retro-projected screens (frontal, right, and left screens) and a direct-projection screen (floor screen). Four infrared cameras allow to monitor any movements that are made from the 3-D glasses. The depth information is encoded in the virtual scene of the CAVE and is restored and conveyed to the eyes by using the 3-D glasses. The user feels like he is moving around the object 360° thanks to different images and angles. As the user’s head moves, the image rotates with it, almost in real-time. This technology guarantees a real life-like experience.

On top of every TMS session, patients will be immersed in the Cave Automatic Virtual Environment (CAVE) wearing 3-D glasses at IRCCS Istituto Auxologico Italiano (see Figure 1 for details). In this fully immersive environment they will be exposed to cues associated with binge eating (i.e., highly palatable food) aimed at extinguishing craving, breaking the bond between the food eliciting craving and binge response (Bernabé et al., 2013) in a virtual scenario linked to binging habits (kitchen, cafeteria, restaurant, and dining room). Patients will be asked to explore and handle food with the joypad.

Further, giving that food craving and anxiety are associated with behavioral changes, physiological data (e.g., HR and HRV) will be assessed during the entire intervention. During the VR exposition, patients will be also required to control and monitor their physiological activation exploiting biofeedback technique. Once physiological indexes get back to a normality range, the VR session will be concluded.

Frequency and severity of BE episodes by means of Bulimia Subscale of Eating Disorders Inventory-3 (EDI-3; Garner, 2004); food craving by means of State-Trait Food Craving questionnaire (FCQ; Cepeda-Benito et al., 2000) and anxiety by means of State-Trait Anxiety Inventory (STAI-Y; Spielberger et al., 1970) will be assessed before and after TMS + VR interventions.

## Discussion

The combination of a neurostimulation technique with a simulative technology would presumably provide better improvement, compared to a single intervention, because it implies the joint presence of neuropsychological and cognitive-behavioral techniques. Transcranial magnetic stimulation treatment might produce a beneficial effect in food addiction, in conjunction with a new promising technology that have become widely used in the clinical setting, such as VR.

On one hand, we expect that TMS will attenuate anxiety, urge to eat and food craving to several studies which showed its potential in reducing addictive symptoms and in restoring neural homeostasis in SUD (Koob and Volkow, 2016; Steele et al., 2019) and in ED disorders (Grall-bronnec and Sauvaget, 2014). In fact, in food addiction, similarly to SUD, reduced neural activity of DLPFC and basial ganglia determine decreased control and decision-making skills.

According to previous studies (Riva et al., 2019a), watching high-palatable food in a virtual scenario linked to binging habits is expected to elicit strong emotions, anxiety, food craving, impulse to over-eat, and guilt feelings like those elicited by real food. At the same it should determine the parasympathetic nervous system activation which can be detected by several physiological indices (e.g., HR and skin conductance) (Koskina et al., 2013; Pla-sanjuanelo et al., 2015). Since patients may experience and increase in HR and skin conductance in the VR scenario we aim to investigate first if TMS (active/sham) has an effect on these parameters; secondly, if these parameters decrease with habituation to the scenario and how long it takes.

More, another VR potential consists in reproducing life-like behaviors in an ecological and controlled setting. This exposure therapy aims not only to break the bond between the food eliciting craving and binge response but also to help patients to recognize and monitor symptoms thanks to biofeedback. In fact, biofeedback by providing real-time feedback about their physiological data, teaches patients to change or self-regulate their physiological activity (Arns et al., 2016). In fact, HRV is related to emotional regulation and appears decreased in craving behavior (Rodríguez-Ruiz et al., 2009, 2012). Meule and colleagues (Meule and Gearhardt, 2014) showed that individuals with craving and overeating behaviors reported a significant decrease in preoccupation with food, lacking control and feeling of guilty after HRV biofeedback.

Therefore, anxiety self-reported symptoms, food craving and binge eating episodes are expected to reduce after TMS + VR-CET intervention.

Overall, it is plausible to suggest that this neuromodulation and cognitive-behavioral techniques will give rise to more effective treatments for food addiction. On one hand, TMS involves neuroplasticity in lateral prefrontal regions; on the other hand, VR targets emotional and behavioral monitoring and management.

## Conclusion

The effects of certain foods on the brain make it hard for some people to avoid them. Food addiction is an addiction to high palatable food, and it has been compared to drug addiction, involving same reward brain areas and neurotransmitters like dopamine. Its prevalence appears increased in patients who suffer from BN and BED. Although several advances in understanding the neural substrates underlying this disease have been made, concomitant improvements in therapies are lacking.

We are proposing an intervention that may reduce craving in patients with food addiction and consequent EDs. This innovative approach would be based on both neurostimulation by rTMS and exposure to fully immersive VR environments. The high-frequency rTMS over the left DLPFC is expected to provide more neural resources by increasing cortical activity and its integration with VR would allow to improve the management of the emotional and behavioral component of craving.

The strength of this approach is represented by the combination of the effects of rTMS and VR. rTMS potential lies in the ability to improve activation and efficiency of prefrontal areas, while VR provides an ecological setting in which patients could learn to self-monitor their reactions to food with the supervision of a clinician. Furthermore, on one hand tracking physiological parameters allows patients to learn changing or self-regulating their physiological activity by providing real-time feedback; on the other hand, it allows to investigate connections between neural-cognitive changes and physiological activity. Considering that TMS effectiveness in treating EDs is not always clear while VR potential in treating EDs has been repeatedly shown (Riva et al., 2019a), we expect that VR could strengthen rTMS effects by increasing cortical excitability.

A study based on this approach may have some limitations. Some users might not be able to complete the intervention due to the occurrence of cybersickness during and after VR sessions (LaViola, 2000) or the occurrence of discomfort and headache induced by the TMS (Rossi et al., 2009). Furthermore, the uncertainty of the neural mechanism underpinning EDs and the unclear beneficial effects of TMS on EDs might influence the effectiveness of this integrated approach.

This novel approach based on the use of two synergistic interventions with high-end technologies might result in a new potential approach for the management and treatment of food addiction in ED.

## Author Contributions

CS-B and VM conceived, defined, and wrote the first draft of the manuscript. GR supervised the study. All authors revised the final version of the manuscript.

## Conflict of Interest

The authors declare that the research was conducted in the absence of any commercial or financial relationships that could be construed as a potential conflict of interest.

## Footnotes

1 The Allocentric Lock Theory suggests that ED may be associated with impairment in the ability to update a stored, negative allocentric (offline) representation of one’s body with real-time (online/egocentric) perception.
